# Molecular analysis of low‐level mosaicism of the *IKBKG* mutation using the X Chromosome Inactivation pattern in Incontinentia Pigmenti

**DOI:** 10.1002/mgg3.1531

**Published:** 2020-10-21

**Authors:** Miki Kawai, Takema Kato, Makiko Tsutsumi, Yasuko Shinkai, Hidehito Inagaki, Hiroki Kurahashi

**Affiliations:** ^1^ Division of Molecular Genetics Institute for Comprehensive Medical Science Fujita Health University Toyoake Japan; ^2^ Department of Clinical Genetics Fujita Health University Hospital Toyoake Japan

**Keywords:** *IKBKG*, incontinentia pigmenti, mosaicism, X chromosome inactivation

## Abstract

**Background:**

Incontinentia pigmenti (IP) is a rare X‐linked disorder affecting the skin and other ectodermal tissues that is caused by mutation of the *IKBKG*/*NEMO* gene. Previous studies have reported that the overall mutation detection rate in IP is ~75%. We hypothesized that a low‐level mosaicism existed in the remaining cases.

**Methods:**

Genomic variations in the *IKBKG* gene were examined in 30 IP probands and their family members. Standard mutational analyses were performed to detect common deletions, nucleotide alterations, and copy number variations. To assess skewing of the X chromosome inactivation (XCI) pattern, a HUMARA assay was performed. We compared the results of this analysis with phenotype severity.

**Results:**

Pathogenic variants were identified in 20 probands (66.7%), the rate of detection was suboptimal. The remaining 10 probands tended to manifest a mild phenotype with no skewed X chromosome inactivation that is generally observed in IP patients. Quantitative nested PCR and digital droplet PCR were performed for the 10 patients and mosaicism of the common *IKBKG* deletion were identified in five patients.

**Conclusion:**

Overall, we detected 25 *IKBKG* mutations (83.3%). Determination of the XCI value in advance of mutational analyses for IP could improve the mutation detection rate. Our improved detection rate for these mutations, particularly those with a low‐level mosaicism, may present opportunities for appropriate genetic counseling.

## Graphical abstract

In our study, genomic variations in the *IKBKG* gene were examined in 30 IP probands and their family members. Standard mutational analyses identified pathogenic variants in 20 probands. The remaining 10 probands tended to manifest a mild phenotype with no skewed X chromosome inactivation. Quantitative nested PCR and digital droplet PCR identified an additional five patients with mosaicism of the common *IKBKG* deletion. Overall, we detected 25 *IKBKG* mutations.

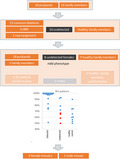

## 1 INTRODUCTION

Incontinentia pigmenti (IP; MIM #308300) is a rare X‐linked genodermatosis with an estimated prevalence at birth of 1.2/100,000 (Orphanet, 2018). This disorder affects the skin and other ectodermal tissues including the eyes, teeth, hair, nail, and central nervous system (CNS). Skin lesions are the first diagnostic manifestations of IP that appear in the neonatal period and comprise a vesicubullous stage (stage I) followed by a verrucous stage (stage II) persisting for months to years, a hyperpigmented stage (stage III), and finally a hypopigmented stage (stage IV) usually continuing throughout life. These skin defects follow Blaschko lines and present in almost all IP patients, thus constituting the principal diagnostic IP criteria for this condition (Landy & Donnai, 1993; Minić et al., 2014). Systemic involvement includes ocular and neurologic impairment. Although IP is X‐linked dominant and is usually lethal in males, some affected males have been reported, representing approximately 10% (Fusco et al., 2014) of the patient population. Affected males present with somatic mosaicism and in some reported cases a concomitant diagnosis of Klinefelter syndrome (Conte et al., 2014).

IP is caused by a mutation of the *IKBKG*/*NEMO* gene (Inhibitor of Kappa polypeptide gene enhancer in B‐cells, Kinase Gamma/Nuclear Factor κB, Essential Modulator, GenBank NM_003639.3, OMIM#300248). This gene encodes NEMO/IKKγ which is required for the activation of the nuclear factor‐kappa B (NF‐κB) transcription factor. NEMO/IKKγ acts as a regulatory subunit of the inhibitor of the κB (IκB) kinase (IKK) complex. The absence of NEMO/IKKγ protein renders the cells sensitive to apoptosis, leading to lethality in males (Aradhya, et al., 2001) and selective skewed X‐inactivation in females (Fusco et al., 2004). In most patients, IP onset is due to loss‐of‐function (LoF) mutations, although in some case *IKBKG* hypomorphic mutations have been reported (Fusco et al., 2008) that reduce but do not eliminate NF‐κB activation, thus explaining why some affected male patients survive (Döffinger et al., 2001). Female patients carrying these hypomorphic mutations show mild signs of IP (Aradhya, et al., 2001).

*IKBKG* is a 23‐kb gene composed of nine coding exons, four alternative noncoding first exons (from exons 1A to 1D), and two promoters. The *IKBKG* gene also shares a part of a 35.7 kb segmental duplication arranged in the opposite direction, one covering the genuine gene and the other a part of a pseudogene copy. The non‐functional *IKBKGP* spans the region between exon 3 to 10. The most frequent IP mutation is a recurrent deletion produced by non‐allelic homologous recombination (NAHR) due to a misalignment between approximately two 650 bp short interspersed nuclear element (SINE) of a medium reiterated 67B (MER67B), one of which is located in intron 3 and the other distally to exon 10. This deletion removes exons 4‐10, spanning 11.7 kb. In addition, the NAHR mechanism can also generate benign copy number variations (CNVs) through an exon 4‐10 deletion in *IKBKGP* or an exon 4‐10 duplication in the *IKBKG* gene. However, both benign CNVs are risk alleles for de novo generation of a pathological *IKBKG* exon 4‐10 deletion (Fusco et al., 2009). The *IKBKG* region contains many repeat sequences and is considered to be prone to rearrangement.

Previous studies have reported that the overall mutation detection rate in IP is 77.6%. *IKBKG* gene targeted analysis of the exon 4‐10 deletion can detect 65% of affected patients, single‐nucleotide variant (SNV) analysis can detect 8.6%, and large duplication and deletion analysis can detect 4% of clinically affected individuals (Fusco et al., 2008; Fusco et al., 2007; Fusco et al., 2012). As *IKBKG* is the only gene responsible for IP, these rates are low in comparison with other genetic diseases, even when taking into consideration that the *IKBKG* locus has a complex genomic structure. In the course of the mutational analyses of our own IP patients, we observed that patients with no detectable mutation showed a mild phenotype. We hypothesized that a low‐level mosaicism existed in these cases which would have made it difficult to detect the mutations in blood samples.

In this study, we conducted *IKBKG* gene mutational analysis in 30 IP patients. We compared the results of this analysis with phenotype severity and also with the X chromosome inactivation (XCI) pattern that would not appear skewed in patients with mosaicism due to the presence of normal cells presenting with a random XCI pattern. We describe observed relationships between phenotype severity, XCI pattern and somatic mosaicism. We also discuss the clinical implications of our results for future genetic testing and counseling.

## 2 MATERIALS AND METHODS

### 2.1 DNA samples

All research was performed in accordance with the principles of the Declaration of Helsinki, and the Ethical Guidelines for Human Genome/Gene Analysis Research by the Ministry of Education, Culture, Science, and Technology, the Ministry of Health, Labor, and Welfare, and the Ministry of Economy, Trade, and Industry of Japan.

Peripheral blood samples were obtained from 30 Japanese IP patients (28 females) in accordance with the research protocol approved by the local ethics committee of Fujita Health University. The IP patients enrolled in our study were selected on the basis of the previous Landy and Donnai (Landy & Donnai, 1993) diagnostic criteria, and also because they met the recently updated criteria (Minić et al., 2014). The severity of disease in each patient was evaluated using manifestation score of skin leision, nervous system defect, ocular system defect, dental system defect, hair defect, and nail defect (Table S1). We collected DNA samples from both parents in 10 cases and a single parent in six cases. Written informed consent to participate in the study was obtained from the patients or their parents. Genomic DNA from peripheral blood was extracted using a conventional salt precipitation technique.

### 2.2 Analysis of common Incontinentia Pigmenti deletions

Common IP deletions have previously been characterized (Bardaro et al., 2003). Long‐range PCR was performed in our current analysis to detect the specific *IKBKG* exon 4‐10 deletion using the primers listed in Table S3. The PCR reactions were performed in 25 μl volumes containing 1 μl of sample, 2.5 U of LA Taq Polymerase (Takara Bio Inc.), LA Taq buffer II, 200 μM dNTPs, 2 mM MgCl_2_ and 10 pmol of each primer. The amplification protocol comprised 30 cycles of 10 seconds at 98°C and 3 min at 65°C. To detect *IKBKGP*, PCR was performed as described above with the previously reported Rev‐2 and JF3R primers (Bardaro et al., 2003).

Nested PCR was performed to detect low‐level mosaic deletions. The primers used for these second amplifications are also listed in Table S3. The resulting PCR products were diluted 1:100 with Tris‐EDTA Buffer (TE). These nested reactions contained 1 μl of template DNA in an identical reaction mixture for 20 cycles of 10 seconds at 98°C and 1 min at 60°C. PCR products were visualized on a 2% agarose gel, quantified using an image analyzer, and compared with the products amplified from serial dilutions of genomic DNA from patients harboring the common *IKBKG* deletion.

To detect mosaic deletions, we performed droplet digital PCR using the custom probes listed in Table S4. Droplet generation, PCR cycling, and droplet reading were performed in accordance with the manufacturer's protocol (Bio‐Rad). The first PCR was performed over 13 cycles with 10 ng of DNA sample as the template. The resulting PCR products were diluted 1:10 with Tris‐EDTA Buffer. Probes and primers were mixed with a 2x ddPCR supermix and with 1 μl of template DNA. In total, 22 μl volume reactions were loaded into an 8‐channel droplet generator cartridge (Bio‐Rad) and droplets were then generated with 70 μl of droplet generation oil (Bio‐Rad) using a manual QX200 Droplet Generator. Following droplet generation, samples were transferred to a 96‐well PCR plate, heat sealed and then amplified on a thermal cycler using the following cycling conditions: 95℃ for 10 min followed by 40 cycles at 95℃ for 30 seconds and 60℃ for 10 min, one cycle at 98℃ for 10 min, and maintenance at 12℃. Post‐PCR products were analyzed on the QX200 droplet reader (Bio‐Rad) using QuantaSoft software.

### 2.3 Analysis of structural variants

Copy number variations were analyzed at the IP locus, including the *IKBKG* gene and *IKBKGP*, by applying the SALSA MLPA probemix P073‐A1 (MRC‐Holland). Test fragments have been designed previously to evaluate the *IKBKG* gene and the sizes and locations of most of these test probes as well as reference probes have been defined. MLPA and capillary electrophoresis‐based amplification product separation (ABI3130, Life Technologies) was performed in accordance with the manufacturer's instructions. Relative copy numbers were obtained after normalization of the peaks against blood‐derived controls. Sequences were analyzed using Gene mapper software.

### 2.4 Analysis of nucleotide alterations

To detect SNVs within *IKBKG* coding sequences, the coding exons were amplified using nested PCR without amplification of *IKBKGP*. Electropherograms were then obtained by Sanger sequencing using Big Dye Terminator Cycle Sequencing Reactions on an ABI 3100 device (PE Applied Biosystems) and were compared with those for genomic sequences from control samples. To evaluate the low‐level mosaicism of the SNVs, we performed deep sequencing of the *IKBKGP* region using next generation sequencer (NGS). We performed long‐range PCR for three regions including the entire *IKBKG* gene and pseudogene. The primers used are listed in Table S5. Three PCR reactions were performed under the following cycling conditions: an initial denaturation of 94℃ for 2 min followed by 5 cycles of 98℃ for 10 s and 74℃ for 5 min, 5 cycles of 98℃ for 10 s and 72℃ for 5 min, 5 cycles of 98℃ for 10 s and 70℃ for 5 min, and 20 cycles of 98℃ for 10 s and 68℃ for 5 min. Successful amplification of the three PCR products was confirmed by agarose gel electrophoresis. Pooled DNA libraries were prepared using a Nextera XT DNA Sample Preparation Kit in accordance with the manufacturer's protocol (Illumina). Paired ends were sequenced for 150 bp using a Miseq Reagent Kit v2 (Illumina). The sequence reads were aligned with the human genome (hg38) reference sequence after manual editing to use between chrX:154541001‐154567000 with BWA aligner. Approximately 10,000 reads from a single patient were obtained and analyzed for genotyping. Downstream processing was carried out with the Genome analysis toolkit (GATK), SAMtools (http://samtools.sourceforge.net/) and Picard Tools (http://broadinstitute.github.io/picard/). Substitution and Indel calls were made with a GATK HaplotypeCaller and GenotypeGVCF (Boisson et al., 2019). Approximately 10,000 reads from a single patient were obtained and analyzed for genotyping.

Variants that have not been registered in any database, are submitted as a new variant in Leiden Open variation Database (http://www.lovd.nl/3.0/home).

### 2.5 X chromosome inactivation studies

To examine the X chromosome inactivation (XCI) pattern, we performed a HUMARA assay according to a previously described protocol (Beever et al., 2003) to assess skewing of the X chromosome inactivation. We first digested the genomic DNA with the methylation‐sensitive restriction enzyme *Hpa*II. PCR primers, one of which was labeled with FAM, were designed across the polymorphic CAG repeat and two *Hpa*II sites in the androgen receptor (*AR*) gene on the X chromosome. In addition to the *AR* locus, the *SLITRK4* and *PCSK1N* loci were used in case the *AR* locus was not informative (Bertelsen & Tümer, 2011). PCR amplification would be achieved only from the inactivated allele harboring methylated *Hpa*II sites. PCR products were analyzed by capillary electrophoresis (ABI3730 Genetic Analyzer) and quantified via the area under the curve using GeneMapper software. A skewed XCI was determined when the inactivated allele was biased at more than 90% (Beever et al., 2003).

### 2.6 Statistical analysis

Intergroup comparisons were made using the Student's *t*‐test or one way analysis of variance method and *P* values of less than 0.05 were considered statistically significant.

## 3 RESULTS

**FIGURE 1:**
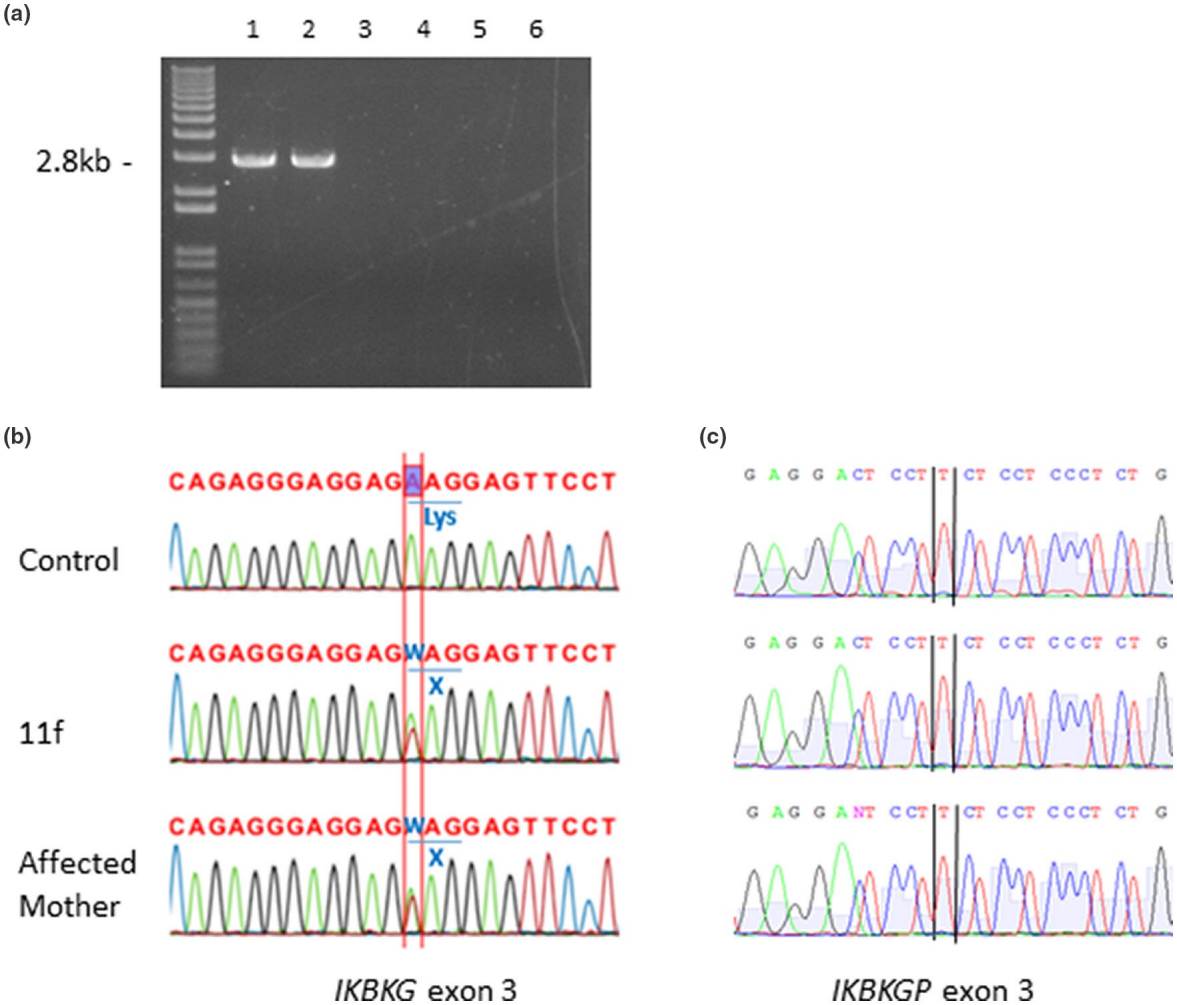
Mutational analysis of the *IKBKG* gene in the IP study patients. (a) PCR analysis of the IP patient blood samples. Lane 1, *IKBKG* deletion exon 4‐10 control. Lane 2, patient 18f. The *IKBKG* deletion was detected. Lanes 3 and 4 are the father and mother of 18f. Lanes 5 and 6 are a healthy control and distilled water (DW). (b) Sanger sequencing of patient 11f and her affected mother. Coding exons for the *IKBKG* gene were amplified by PCR, being not amplified together with IKBKGP.NM_001099857.2:c.268A>T, NP_001093327.1: p.Lys90X is confirmed. (c) Pseudogene‐specific sequencing. Sequencing of 11f and her mother did not show the pathogenic variant at chrX:154648193 on *IKBKGP*.

In our current IP cohort, we detected the *IKBKG* exon 4‐10 common deletion in 13 patients (03f, 05f, 06f, 08f, 13f, 14f, 16f, 18f, 21f, 22f, 28f, 29f, and 30f; Figure 1a) which was a prevalence (detection rate) of 43.3％ among the total series of 30 patients (Table 1). Among these 13 patients with the common deletion, three were familial cases (03f, 14f, 16f) and we confirmed in one case that the same deletion was present in the mother (03f). We next performed Sanger sequencing in the 17 IP patients who did not carry the recurrent deletion. Pathogenic SNVs were detected in 6 cases [07f, c.343A>T (p.K115X); 09f, c.896delC (p.P299RfsX3); 11f, c.268A>T (p.K90X; Figure 1b,c); 17f, c.184C>T (p.R62X); 23f, c.913‐2A>G; 26f, c.976_978delAAG (p.K326del)]. These identified mutations included three nonsense mutations (07f, 11f, 17f), one frameshift mutation (09f), one in‐frame amino acid deletion (26f), and one splicing mutation (23f). To identify structural rearrangements caused by large deletions or duplications at the IP locus, including *IKBKG* and neighboring genes, we performed MLPA analysis to investigate copy number variation in the region. We thereby identified a large deletion including the *IKBKGP* pseudogene (10f). Although we identified pathogenic variants in 20 of the 30 study patients (66.7%), this rate of detection was suboptimal.

**TABLE 1:** Summary of the mutation.

Patient	Age at genetic testing	Female/male	Mutation^a^	Exon/intron	Inheritance	Phenotype^b^ Score	XCI pattern
01f	0	F	ND	—	Sporadic	2	91.8%
02f	3	F	ND	—	Sporadic	2	79.0%
03f	0	F	ex 4‐10 deletion	ex 4‐10	Familial	4	91.3%
04m	0	M	ND	—	Sporadic	2	(male)
05f	0	F	ex 4‐10 deletion	ex 4‐10	Sporadic	3	NI
06f	0	F	ex 4‐10 deletion	ex 4‐10	Sporadic	3	99.0%
07f	0	F	c.343A>T (K115X)	ex 3‐10	Familial	3	82.4%
08f	3	F	ex 4‐10 deletion	ex 4‐10	Sporadic	3	100.0%
09f	0	F	c.896delC (P299RfsX3)	ex 7‐10	Familial	4	99.1%
10f	0	F	94 kb deletion	ex 3‐10	Familial	4	97.3%
11f	0	F	c.268A>T (K90X)	ex 3‐10	Familial	3	98.4%
12f	2	F	Mosaic ex 4‐10 deletion	ex 4‐10	Sporadic	2	67.1%
13f	0	F	ex 4‐10 deletion	ex 4‐10	Sporadic	3	100.0%
14f	48	F	ex 4‐10 deletion	ex 4‐10	Familial	5	100.0%
15f	0	F	ND	—	Sporadic	2	72.2%
16f	0	F	ex 4‐10 deletion	ex 4‐10	Familial	3	100.0%
17f	0	F	c.184C>T (R62X)	ex 2	ND	3	97.4%
18f	0	F	ex 4‐10 deletion	ex 4‐10	Sporadic	5	NI
19f	0	F	Mosaic ex 4‐10 deletion	ex 4‐10	Sporadic	1	50.0%
20f	0	F	Mosaic ex 4‐10 deletion	ex 4‐10	Sporadic	1	58.4%
21f	2	F	ex 4‐10 deletion	ex 4‐10	Sporadic	3	100.0%
22f	34	F	ex 4‐10 deletion	ex 4‐10	ND	3	100.0%
23f	44	F	c.913‐2A>G (p.spl)	int 7	Familial	5	100.0%
24m	0	M	Mosaic ex 4‐10 deletion	ex 4‐10	Sporadic	2	(male)
25f	0	F	Mosaic ex 4‐10 deletion	ex 4‐10	Sporadic	2	65.3%
26f	2	F	c.976_978delAAG (K326del)	ex 8	ND	3	75.7%
27f	33	F	ND	—	Sporadic	2	92.8%
28f	30’s	F	ex 4‐10 deletion	ex 4‐10	ND	5	98.5%
29f	1	F	ex 4‐10 deletion	ex 4‐10	Sporadic	3	51.3%
30f	1	F	ex 4‐10 deletion	ex 4‐10	Sporadic	3	98.3%

Note

Homozygote for the polymorphic CAG repeat in the *AR* gene.

Abbreviations: ND, not determined. NI, not informative.

^a^ The mutation numbering is based on the IKBKG cDNA sequence according to the GenBank Accession number NM_003639.3. Codon numbering starts from the translation initiation codon 1 according to the GenBank Accession number NP_003630.1.

^b^ Phenotype score was allocated from Table S2.

**FIGURE 2:**
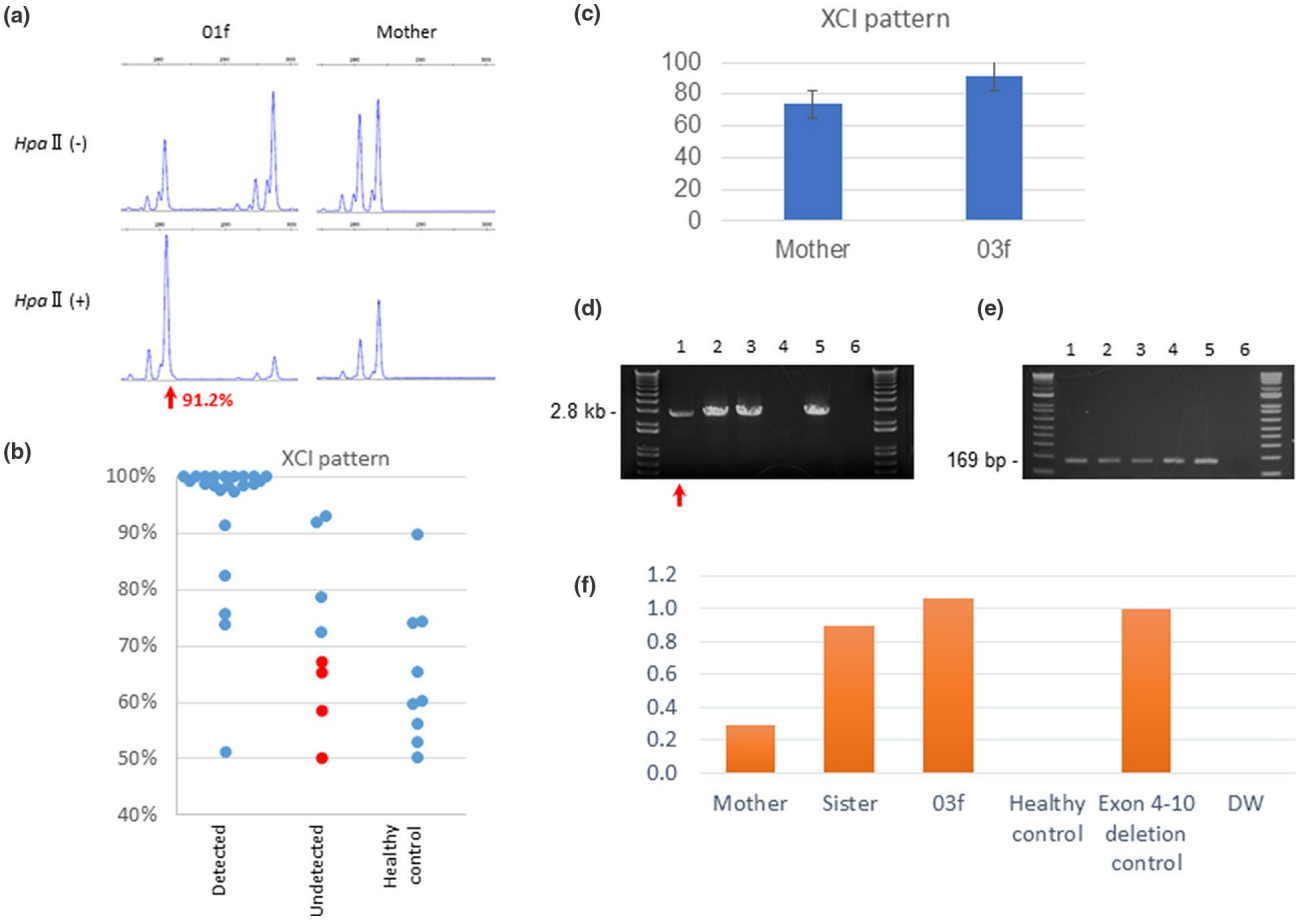
XCI patterns among IP patients in the study cohort. (a) Electropherogram of the HUMARA assay data showing the fragment analysis of PCR products amplified from undigested and digested DNA of patient 01f and her mother. The two major peaks represent two alleles with different numbers of short tandem repeats at the HUMARA locus (red arrows). After digestion, the DNA of the mother displayed a matching pattern of preferential loss of the short alleles. (b) Scatter plot of the informative XCI pattern of female IP probands and their mothers. “Detected” represents 18 probands, 4 carrier mothers, and 1 carrier sisters in whom an *IKBKG* mutation was detected. The median XCI value was 98.5% in these 23 cases. “Undetected” represents 8 probands with a median XCI value of 69.7%. The median XCI value of the healthy controls was 60.2%. Mosaic mutations are denoted by red dots. 11 probands, 1 carrier mother and 1 carrier sister carried the exon 4‐10 deletion; one patient harbored a 94 kb deleted rearrangement; six probands and their three carrier mothers harbored SNVs; eight female probands had unknown mutations. The median age of the patients was 0 years and of the healthy controls was 40 years. (c) Median XCI values at the *AR*, *ZDHHC15*, *SLITRK4*, and *PCSK1N* loci. A lower XCI level in the mother may indicate somatic and germline mosaicism. (d) Common deletion‐specific PCR analysis. 2.8 kb PCR products indicate the common exon 4‐10 deletion of the *IKBKG* gene. Lanes 1‐3 represent the mother of 03f, the sister of 03f and 03f, respectively. Lane 4 is a healthy control. Lane 5 is an *IKBKG* exon 4‐10 deletion control and lane 6 is DW. (e) Internal standard for semi‐quantitative PCR. The same DNA samples were used as templates for control PCR to amplify intron 45 of the *DMD* gene. (f) Semi‐quantitative PCR. Using image analyzer, measurements of deletion specific PCR products were divided by that of internal control PCR products (lane 5). Mother of 03f is likely to have a mosaic deletion since the amount of the PCR products was less than that of 03f and her sister with heterozygous deletion.

In the remaining 10 patients with no detectable mutations via standard analyses, we observed that their skin symptoms were less severe than the patient with detectable *IKBKG* mutations. In the evaluations of phenotype severity using our scoring system, the detected mutation cases had a score of 3.55 (n = 20), and those without a detectable mutation had a score of 1.8 (n = 10), which was significantly low (*p* < 0.01; Table 1, Table S1). To exclude the possibility that these patients had another autosomal disease that mimics IP, their XCI status was analyzed using a HUMARA assay since female X‐linked IP patients generally manifest a selective skewed X‐inactivation. We performed the HUMARA assay on all of the female patients in our current IP cohort (n = 28) as well as some of their female family members (n = 16; Figure 2a, Table S2). We could not however determine the allelic status in some of the cases since the polymorphic CAG repeat in the *AR* gene was homozygous in five of the women, including the mother of 03f, whose values of XCI pattern was determined by the other method (described later). We obtained the values of XCI pattern of 40 females (31 patients and 9 unaffected family members).

Among the informative 31 patients, the skewed XCI values ranged from 50% to 100%, indicating that some patients showed a selectively skewed XCI pattern while others showed a random XCI pattern. We divided the patients into two categories: patients with an already detected or undetected *IKBKG* mutation. The mean value for the XCI patterns of the patients with a detected mutation was 98.5% (n = 23). In contrast, the XCI pattern values of the patients without a detected mutation (n = 8) ranged from 50% to 92.8% (mean 69.7%), which was significantly low (*p* < 0.01; Figure 2b, Table S2). The mean value for the unaffected family members was 60.2% (n = 9; Figure 2b). It is possible that our remaining 10 patients without a detected mutation and with a mild IP phenotype might have had a disorder other than X‐linked IP. However, one of our patients with an in‐frame deletion of a single amino acid (p.K326del) in the *IKBKG* gene (26f) had an XCI value of 75.7%, indicating that a hypomorphic variant does not lead to a selectively skewed XCI. Although the K326 residue in the *IKBKG* gene product is located at a linear polyubiquitinated site (Ikeda et al., 2011) and is an evolutionarily conserved position, protein products with an in‐frame deletion may possibly have some residual activity that prevents cellular lethality. Hence, it is likely that the patients with a mild phenotype and with an almost random XCI pattern harbor weak variants such as somatic mosaicism.

Further evidence of the relationship between phenotype severity and the XCI pattern was observed in patient 03f and her mildly affected mother, both of whom carried the common *IKBKG* deletion (Table 2). Since the polymorphic CAG repeat in the *AR* gene was homozygous in the mother, we instead assessed three alternative methylation–sensitive restriction enzyme sites in the *ZDHHC15*, *SLITRK4* and *PCSKIN* genes, respectively. The XCI patterns for patient 03f were 89.2% in *SLITRK4*; 93.1% in *PCSKIN*, a median of 91.3%, whereas those for the mother were 67.7% in the *ZDHHC15* locus; 73.3% in *SLITRK4*; 82.5% in *PCSKIN*, a median of 73.7% (Figure 2c, Table 3). These data indicated that patient 03f manifests a selective skewed pattern, but that her mildly affected mother does not. Retrospectively, the amount of deletion‐specific PCR products in the mother was less than that of the proband (Figure 2d). This finding indicated that the mother, who had a mild phenotype, may have somatic mosaicism of the common *IKBKG* deletion.

**TABLE 2:** Phenotype score of the 03f family.

Patient	Age at genetic testing	Female/male	Skin leision^a^	Nervous system defect^b^	Ocular system defect^c^	Dental system defect^d^	Hair defect^e^	Nails defect^f^	Phenotype score^g^
03f (proband)	0	F	3	1	ND	ND	ND	ND	4
Sister of 03f	3	F	3	0	0	0	1	0	4
Mother of 03f	38	F	2	0	0	0	0	0	2

Note

IP phenotype score analysis. A phenotype score of clinical severity was derived when possible in IP patients whose clinical data were available.

Abbreviation: ND, not determined.

Phenotype score represents the addition of the single score values for each system/organ.

^a^ We assigned a score 1 for little skin abnormality in limbs; a score 2 for skin abnormality in some parts of limbs or trunk; a score of 3 for skin abnormality in all limbs and trunk. All IP reported patients suffered skin abnormality.

^b^ A score of 1 was added for each nervous system (NS) defect (seizures, or spastic paresis, or motor retardation, or mental retardation or microcephaly).

^c^ A score of 1 was added for each ocular system defect (strabismus, or cataracts, or optic atrophy, or retinal vascular pigmentary abnormalities, ormicrophthalmos, or pseudogliomas).

^d^ A score of 1 was added for each Dental System defect (partial anodontia, or delayed dentition, or cone/peg shaped teeth, or impactions).

^e^ A score of 1 was added for each Hair defect (vertex alopecia, or wooly hair nevus, or eyelash and eyebrow hypogenesis).

^f^ A score of 1 was added for each Nails defect (onychogryposis, or pitting, or ridging).

^g^ Phenotype score represents the addition of the single score values for each system/organ.

**TABLE 3:** XCI value of AR and three alternative methylation‐sensitive restriction enzyme site.

	*AR*	*ZDHHC15*	*SLITRK4*	*PCSKIN*	Median (%)
03f	91.3	NI	89.2	93.1	91.3
Sister of 03f	98.3	—	—	—	98.3
Mother of 03f	NI	67.7	73.7	82.5	73.7

Abbreviation: NI, not informative.

This means that the patients who are positive in PCR for common *IKBKG* deletion might possibly include those with somatic mosaicism. We reanalyzed the PCR‐positive patients (03f, 05f, 06f, 08f, 15f, 17f, 28f, 29f, 30f, mother of 03f) by less sensitive MLPA. MLPA detected the common *IKBKG* deletion in all patients except for 29 f. Although the possibility of the effect of duplication polymorphism has not been ruled out, this results suggest that 29f might also harbor somatic mosaicism since the XCI pattern of 29f (51.3%) support this speculation.

**FIGURE 3:**
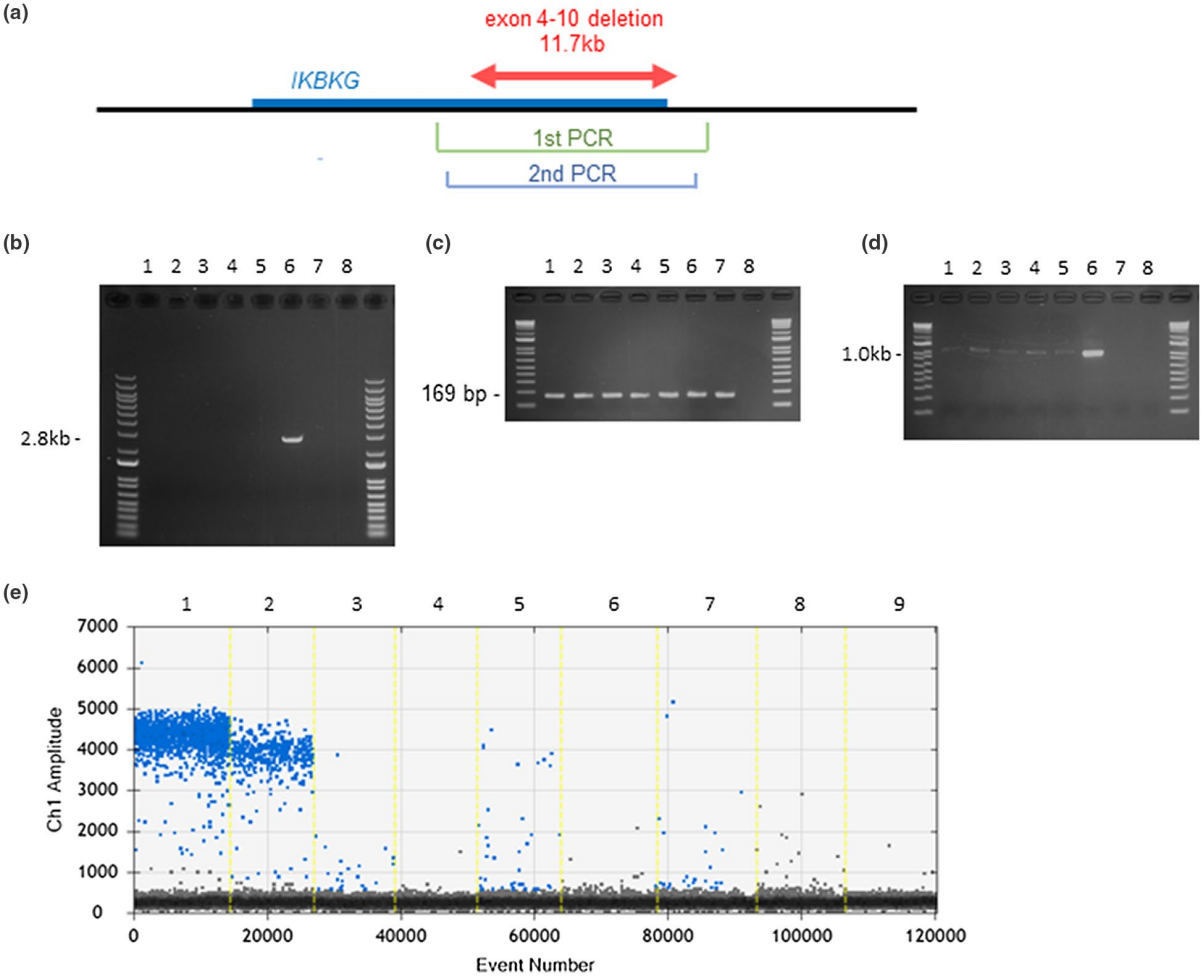
Mosaic analysis of the *IKBKG* gene. (a) Schematic showing the relative location of the *IKBKG* gene and its exons 4‐10. The position of the common exon 4‐10 deletion in IP is indicated by red arrows. The green line denotes the region of the first PCR and the blue line the second PCR. (b) First PCR. A 2.8 kb amplified product indicates an *IKBKG* exon 4‐10 deletion. Lanes 1‐5 represent 12f, 19f, 20f, 20m, and 25f, respectively. Lane 6, *IKBKG* exon 4‐10 deletion control; lane 7, healthy control; lane 8; DW. (c) Internal standard for semi‐quantitative PCR. The same DNA samples were used as templates for control PCR to amplify intron 45 of the *DMD* gene. (d) Second PCR. A 1.0 kb amplified product indicates a mosaic deletion, which was evident in lanes 1‐5. Lanes 6‐8 are as described in (b). (e) ddPCR. Plots show a quantification of fluorescence signal of droplet. Green dots indicate fluorescence‐positive droplets, while black dots indicate negative droplets. Lane 1 represent *IKBKG* exon 4‐10 deletion control. Lane 2, 3, 4, 5, 6, 7 represent the mother of 03f, 12f, 19f, 20f, 24m, 25f, respectively. Lane 8,9 represent healthy control and DW, respectively.

A nested PCR assay in the remaining 10 patients revealed low‐level mosaicisms of the common deletion in five cases (12f, 19f, 20f, 24m, 25f; Figure 3a‐d). Using an image analyzer, the mosaic ratios in 12f, 19f, 20f, 24m and 25f were found to be 1/70, 1/20, 1/43, 1/20, and 1/62, respectively. With the ddPCR assay, the mosaic ratios in those patients were 1/75, 1/57, 1/39, 1/76, and 1/56, respectively (Figure 3e). Except for one male patient, we reviewed the XCI pattern in four of the female IP patients with a mosaic deletion and found XCI patterns of 67.1%, 50%, 58.4%, and 65.3%, respectively, with a mean value of 61.9%. The mean XCI value of the remaining four cases with undetected mutations was 79.0%, compared to 60.2% in the healthy controls. These XCI values thus showed distinct differences. Hence, as the XCI pattern of the mother, who had clinical symptoms, of 03f was 73.3%, this lower XCI may indicate a somatic and germline mosaicism. Our result of PCR assay and ddPCR assay revealed a mosaic ratio of 1/4 and 1/5 for the mother of 03f, respectively (Figure 3e). To confirm the somatic mosaicism, we attempted to obtain somatic tissues other than blood in these patients. Cheek swab sample was obtained only from 24 m, which did not show the common deletion by the nested PCR (data not shown).

We finally performed NGS analysis to detect mosaic SNVs in the remaining subjects (01f, 02f, 04m, and 15f). Approximately, 90% of the cumulative target region was covered with a sequence depth of more than 200X. We evaluate SNVs and indels at a greater than 1% frequency in the coding exons of the *IKBKG* gene and *IKBKGP* pseudogene. No SNVs that indicated low‐level mosaicism were identified in these patients. These data are summarized in Table 1. When including the low‐level mosaic mutations, we detected a total of 25 *IKBKG* mutations in 30 samples (83.3%).

## 4 DISCUSSION

In our current study of 30 unrelated IP families from Japan, we identified *IKBKG* mutations in 25 families, including 5 low‐level mosaic mutations. In a previous large‐scale study of IP, 65% of the cases had the common exon 4‐10 deletion in the *IKBKG* gene (Fusco et al., 2008), 8.6% harbored SNVs (Fusco et al., 2007), and 4% had a larger deletion at the *IKBKG* locus (Conte et al., 2014). In our current standard protocol, we initially identified the common *IKBKG* deletion at a much reduced frequency of 43.3% (13/30), which made our initial mutation detection efficiency low (20/30, 66.7%) in comparison to previous studies. This outcome might have partly been due to ethnic differences. Another possible reason may be that there was a bias in the patients referred to our institute, that is, they were mainly non‐deleted cases that came via deletion screening in a prior facility. We finally identified a total of 25 mutations in 30 IP patients after introducing a detection system for low‐level mosaicism (83.3%).

We identified low‐level mosaicism of the common *IKBKG* deletion in 16.7% of our current IP cases (5/30). Thus, as much as 27.8% of the common deletions in our current study series were mosaic (5/18). This suggested that our diagnosis system, semi‐quantitative nested PCR and ddPCR, could efficiently detect low‐level mosaicism. We conclude from this that clinical geneticists should become aware of the high incidence of low‐level mosaicism in this disorder. Our present data have indicated that IP patients with mild symptoms only affecting the skin and those with low XCI values tended to include cases of low‐level mosaic mutations in the *IKBKG* gene. It is well documented that XCI values are elevated with age (Hatakeyama et al., 2004). Our current patients median age was 0 years old in both groups (no significant difference), although that of the unaffected family members was 38.5 years. Thus, these are likely predictors of a low‐level mosaicism when a mutation survey fails to detect mutations in the *IKBKG* gene. Hence, a HUMARA assay may be a powerful method of differentiating the mutational status of the *IKBKG* gene in IP patients. We recommend the use of this assay when the standard mutational analyses fail to detect mutations in the *IKBKG* gene in IP patients.

It is well acknowledged that random XCI in humans occurs in early embryogenesis after the blastocyst stage (Lee & Bartolomei, 2013). If a zygote with a germinal mutation in the *IKBKG* gene undergoes random XCI, about half of the cells carry the mutation on the activated X chromosome and the other half carry the mutation on the inactive X. During early embryogenesis, the cells with the mutation on the activated X chromosome would undergo apoptosis and be negatively selected. Thus, a skewed XCI pattern would be established. A small number of surviving cells harboring the *IKBKG* mutation on the activated X chromosome would then induce local mild symptoms. In contrast, if the mutation in the *IKBKG* gene arises in the very early embryonic stages prior to XCI determination, most of the cells would be normal and lack the mutation. Even if the cells with the mutation on the activated X chromosome would be negatively selected, XCI data would show a random XCI pattern reflecting a majority of normal cells. Finally, if the mutation in the *IKBKG* gene arises after XCI determination, XCI data would show a random XCI pattern reflecting the majority of normal cells regardless of whether the mutation occurred on the activated or inactivated X chromosome. According to their mosaic ratio, our current study probands were found to have undergone the somatic mutation event at the embryonic stage from the end of the morula stage, which consists of 16‐36 cells, to the blastocyst stage. However, whether these mutations occurred before or after the XCI patterns are determined, patients with low‐level mosaicism would not manifest skewed XCI patterns.

We did not detect any mutations in the *IKBKG* gene in four patients (01f, 02f, 04m, 15f and 27f). One of these cases (27f) showed a skewed XCI pattern (92.8%), suggesting that she had a germline mutation that we could not detect with our methods. Two of our current study patients (02f and 15f) did not show a skewed XCI pattern (79.0% and 72.2%, respectively). The other case (04m) was a male patient with a normal karyotype. These three IP patients may therefore have low‐level mosaic mutations not of the common deletion, but at the nucleotide level. The detection sensitivity of the deep sequencing we used prevented us from identifying mosaic nucleotide mutations. With a read depth of 200‐400, up to a 1% mutation frequency in the coding exons of the *IKBKG* gene could be theoretically detected in spite of the presence of random artifacts such as misincorporations during DNA synthesis. However, a level of mosaicism below 1% could not be detected even when this deep sequencing was performed. Further investigations using ultra deep sequencing might elucidate the mutations of these remaining patients.

IP females have a risk of having an affected baby, with the risk of inheritance generally thought to be 50%, that is, male embryonic lethality and the birth of affected females. However, our current analyses indicate that the apparently affected IP females include those harboring germline mutations and those with a low level of mosaicism. Given that IP females can have low‐level mosaic somatic mutations, the risk of inheritance becomes very small. In sporadic IP cases, a mother with an apparently normal phenotype may have a hidden low level of mosaicism. Among the IP patients who carry the *IKBKG* exon 4‐10 deletion, 65% are sporadic cases (Fusco et al., 2009). In addition, 3.8% of IP offspring with a de novo mutation have parents who shows 1% mosaicism in their blood cells (Rahbari et al., 2015). Clinical symptoms of IP appearing in patients with low‐level mosaic mutations might be too mild to be diagnosed properly. Our current findings suggest that most mothers of sporadic cases may have no germline mutation, but some might show a low level of mosaicism when a highly sensitive mutation search is performed. Furthermore, even when the mother of a child with IP is correctly diagnosed with a low level of mosaicism, they would not necessarily have a small risk of having affected children. The mother of our current study patient 03f showed a 1/4 mosaic mutation, but was a parent of three girls of which two were affected with IP. A noteworthy limitation of our current analysis is that we only have the mosaicism information from blood samples and not from the germinal cells. In any event, a correct diagnosis of germline or somatic mosaicism in relation to IP will facilitate more informed genetic counseling for family planning purposes.

In conclusion, we have here identified five patients with low level of mosaicism of the common *IKBKG* exon 4‐10 deletion that causes IP. These patients manifest a mild IP phenotype only and no skewed XCI pattern, suggesting that XCI pattern values can predict the possibility of mosaicism for this disorder. Determination of the XCI value in advance of mutational analyses for IP could improve the mutation detection rate. Detecting mosaic mutations will also be beneficial for genetic counseling of affected individuals.

## CONFLICT OF INTEREST

The authors declare no competing interests in relation to this study.

## AUTHOR CONTRIBUTIONS

MK carried out the genomic analysis and drafting of the manuscript. TK, MT, and HI was responsible for genomic analysis. YS carried out NGS analysis. HK conceived the study and participated in its design and drafted the manuscript. All authors read and approved the final version of the manuscript and agree with the order of presentation of the authors.

## Supporting information

Supplementary Material

## Data Availability

The datasets used and/or analyzed during the current study are available from the corresponding author on reasonable request.
