# Supplementary material for: Molecular analysis of low‐level mosaicism of the IKBKG mutation using the X Chromosome Inactivation pattern in Incontinentia Pigmenti
Source: Mol Genet Genomic Med. 2020 Oct 21;8(12):e1531. doi: 10.1002/mgg3.1531 (PMC7767561; doi:10.1002/mgg3.1531)
Supplement: Supplementary file 1 — Supplementary Material [file MGG3-8-e1531-s001.docx]

**Table S1. Phenotype score of the probands**

Patient Age at Female Skin Nervous Ocular Dental Hair Nails Phenotype

genetic /Male leision^1^ system system system defect^5^ defect^6^ score^7^

testing defect^2^ defect^3^ defect^4^

01f 0 F 2 0 0 ND^8^ ND ND 2

02f 3 F 1 0 0 1 0 0 2

03f 0 F 3 1 ND ND ND ND 4

04m 0 M 2 0 0 0 0 0 2

05f 0 F 3 0 0 ND 0 0 3

06f 0 F 2 0 0 ND 0 0 3

07f 0 F 3 0 0 0 0 0 3

08f 3 F 3 0 0 ND 0 0 3

09f 0 F 3 1 0 ND 0 0 4

10f 0 F 3 0 1 0 0 0 4

11f 0 F 3 0 0 ND 0 0 3

12f 2 F 3 0 0 ND 0 0 2

13f 0 F 3 0 0 0 0 0 3

14f 48 F 3 0 1 1 0 0 5

15f 0 F 2 0 0 0 0 0 2

16f 0 F 3 0 0 ND ND ND 3

17f 0 F 3 0 0 ND ND 0 3

18f 0 F 3 1 0 ND 1 0 5

19f 0 F 1 0 0 ND ND 0 1

20f 0 F 1 0 0 ND 0 0 1

21f 2 F 3 0 0 ND 0 0 3

22f 34 F 3 0 0 ND ND ND 3

23f 44 F 3 0 1 1 0 0 5

24m 0 M 2 0 0 ND 0 0 2

25f 0 F 2 0 0 ND 0 0 2

26f 2 F 3 0 0 0 0 0 3

27f 33 F 1 0 ND 1 ND 0 2

28f 30’s F 3 0 0 1 1 0 5

29f 1 F 3 ND ND ND ND 0 3

30f 1 F 3 0 0 ND ND 0 3

Sister of 03f 3 F 3 0 0 0 1 0 4

Mother of 03f 38 F 2 0 0 0 0 0 2

IP phenotype score analysis. A phenotype score of clinical severity was derived when possible in IP patients whose clinical data were available.

1. We assigned one point for little skin abnormality in limbs; two points for skin abnormality in some parts of limbs or trunk; three points for skin abnormality in all limbs and trunk. All IP reported patients suffered skin abnormality.
2. One point was scored for each nervous system (NS) defect (seizures, or spastic paresis, or motor retardation, or mental retardation or microcephaly).
3. One point was scored for each ocular system defect (strabismus, or cataracts, or optic atrophy, or retinal vascular pigmentary abnormalities, ormicrophthalmos, or pseudogliomas).
4. One point was scored for each Dental System defect (partial anodontia, or delayed dentition, or cone/peg shaped teeth, or impactions).
5. One point was scored for each Hair defect (vertex alopecia, or wooly hair nevus, or eyelash and eyebrow hypogenesis).
6. One point was scored for each Nails defect (onychogryposis, or pitting, or ridging).
7. Phenotype score represents the addition of the single point values for each system/organ.

8. ND, Not determined.

**Table S2. Summary of XCI pattern**

Patient Age at Female Mutation^1^ Phenotype^2^ XCI

genetic /Male score pattern

testing

Patients with a detected mutation (n=23) 98.5%^3^

03f 0 F ex 4-10 deletion 4 91.3%

Sister of 03f 3 F ex 4-10 deletion 4 98.3%

Mother of 03f 38 F ex 4-10 deletion 2 73.7%^4^

06f 0 F ex 4-10 deletion 3 99.0%

07f 0 F c.343A>T (K115X) 3 82.4%

Mother of 07f ND^5^ F c.343A>T (K115X) ND 98.5%

08f 3 F ex 4-10 deletion 3 100.0%

09f 0 F c.896delC (P299RfsX3) 4 99.1%

Mother of 09f ND F c.896delC (P299RfsX3) ND 100.0%

10f 0 F 94kb deletion 4 97.3%

11f 0 F c.268A>T (K90X) 3 98.4%

Mother of 11f ND F c.268A>T (K90X) ND 100.0%

13f 0 F ex 4-10 deletion 3 100.0%

14f 48 F ex 4-10 deletion 5 100.0%

16f 0 F ex 4-10 deletion ND 100.0%

17f 0 F c.184C>T (R62X) 3 97.4%

21f 2 F ex 4-10 deletion 3 100.0%

22f 34 F ex 4-10 deletion 3 100.0%

23f 44 F c.913-2A>G (p.spl) 5 100.0%

26f 2 F c.976_978delAAG (K326del) 3 75.7%

28f 30’s F ex 4-10 deletion 5 98.5%

29f 1 F ex 4-10 deletion 3 51.3%

30f 1 F ex 4-10 deletion 3 98.3%

Patients with a undetected mutation (n=8) 67.1%^6^

01f 0 F ND 2 91.8%

02f 3 F ND 2 78.6%

12f 2 F (Mosaic ex 4-10 deletion)^7^ 2 67.1%

15f 0 F ND 2 72.2%

19f 0 F (Mosaic ex 4-10 deletion)^7^ 1 50.0%

20f 0 F (Mosaic ex 4-10 deletion)^7^ 1 58.4%

25f 0 F (Mosaic ex 4-10 deletion)^7^ 2 65.3%

27f 33 F ND 2 92.8%

Patient Age at Female Mutation^1^ Phenotype ^2^ XCI

genetic /Male score pattern

testing

Unaffected family (n=9) 60.2%^8^

Mother of 01f ND F - - 65.4%

Mother of 02f 42 F - - 56.0%

Aunt of 03f 37 F - - 50.0%

Mother of 05f ND F - - 74.0%

Mother of 10f ND F - - 74.2%

Mother of 12f ND F - - 89.7%

Mother of 13f 40 F - - 60.2%

Mother of 18f ND F - - 59.6%

Mother of 21f ND F - - 52.8%

Patients with uninformative XCI pattern (n=2)

05f 0 F ex 4-10 deletion 3 NI^9^

18f 0 F ex 4-10 deletion 5 NI

Healthy family with uninformative XCI pattern (n=2)

Mother of 08f ND F - - NI

Mother of 20f ND F - - NI

Male patients (n=2)

04m 0 M ND 2 (male)

24m 0 M (Mosaic ex 4-10 deletion)^7^ 2 (male)

1. 1. The mutation numbering is based on the IKBKG cDNA sequence according to the GenBank Accession number NM_003639.3. Codon numbering starts from the translation initiation codon 1 according to the GenBank Accession number NP_003630.1.
2. Phenotype score was allocated from Table S2.
3. Mean value for the patients with a detected mutation
4. Since the polymorphic CAG repeat in the *AR* gene was homozygous in mother of 03f, three alternative methylation–sensitive restriction enzyme sites in the *ZDHHC15*, *SLITRK4* and *PCSKIN* genes were assessed.
5. ND, Not determined.
6. Mean value for the patients with a undetected mutation
7. Mosaic mutation was not detected on first PCR assay, and the mutation was detected afterward on second PCR assay.
8. Mean value for the unaffected family.
9. NI, Not informative. Homozygote for the polymorphic CAG repeat in the *AR* gene.

**Supplementary Table S3 PCR primers used for *IKBKG* exon 4-10 detection**

Size (bp) Forward primer Reverse Primer

First PCR 14476 GAGGACCAATACCGAGCATC GAATTCGGGAGTCAGCTCGGAGAC

Second PCR 12682 TCCTGTGTGGGAAGTGGATG CACAGGAACCAGCAAGGAGC

**Supplementary Table S4 ddPCR probe used for *IKBKG* exon 4-10 mosaic detection**

ddPCR FAM-CGCAGCCACTCCTTGGAAACGGAAACC-TAMRA

**Supplementary Table S5 PCR primers used for multiplex PCR and NGS**

Size (bp) Forward primer Reverse primer

01f 13995 TGGCTGCGATGCTCTAGTGGGACTTT TGGTCTCACATCGTTGGCCTCAAGCA

11556 CCCTAGGAGTCTGCAGGCGTGTTAGG GGATTTAAGTGCAGCACCCGTGAGCA

9372 GAAGCAGTTCTCCCTGAGAGCCCCAG TGCGGCCATCTGTTTTTGCGTGTGTA

02f 13995 TGGCTGCGATGCTCTAGTGGGACTTT TGGTCTCACATCGTTGGCCTCAAGCA

11483 CCCTAGGAGTCTGCAGGCGTGTTAGG GCACCTGGCTTGAAGAAGGGGAAAGG

9399 GAAGCAGTTCTCCCTGAGAGCCCCAG GTTTGCAGAGTCAGCCGTGTGGAAGG

04m 13995 TGGCTGCGATGCTCTAGTGGGACTTT TGGTCTCACATCGTTGGCCTCAAGCA

11556 CCCTAGGAGTCTGCAGGCGTGTTAGG GGATTTAAGTGCAGCACCCGTGAGCA

9372 GAAGCAGTTCTCCCTGAGAGCCCCAG TGCGGCCATCTGTTTTTGCGTGTGTA

15f 13995 TGGCTGCGATGCTCTAGTGGGACTTT TGGTCTCACATCGTTGGCCTCAAGCA

11483 CCCTAGGAGTCTGCAGGCGTGTTAGG GCACCTGGCTTGAAGAAGGGGAAAGG

9372 GAAGCAGTTCTCCCTGAGAGCCCCAG TGCGGCCATCTGTTTTTGCGTGTGTA
